# Discursive construction of corporate identity through websites: An intercultural perspective on the commercial banks of the United States and China

**DOI:** 10.3389/fpsyg.2022.947012

**Published:** 2022-08-24

**Authors:** Heng Fu, Huifen Zhu

**Affiliations:** School of Foreign Languages, Zhejiang Gongshang University, Hangzhou, China

**Keywords:** corporate identity, intercultural communication, semantic categories, social cultural behavior, Hofstede's cultural dimensions theory

## Abstract

With the assistance of the corpus analysis tool Wmatrix 4.0, this paper analyzes the semantic categories of the top 10 commercial banks of China and the United States to figure out their social-cultural behavior in the Internet business context. It is discovered that both common and distinctive identities were constructed: the common identities include the professional financial service provider, responsible corporation for employees, and relevant communities with environmental and social consciousness, while the distinctive identities are manifested in the communication strategy, style, and persuasion mode: (1) The Chinese Commercial Banks adopted the proactive strategy for corporate identity construction, are prone to take hierarchical and impersonal communication style, and more focused on the “credibility appeal” and “rational appeal” in persuasion mode; (2) the commercial banks of the United States are more reactive in the communication strategy, position themselves in short distance with the putative audience in communication style, and conform to the typical “affective appeal” regarding the persuasion mode. From the intercultural perspective, the distinctions are the representation of the peculiar high-context culture and low-context culture based on Hofstede's cultural dimensions theory. Chinese banks should try to shorten the cultural gap by adopting communication strategy in conformity with the local cultural when going global rather than sticking to the domestic communication strategy.

## Introduction

Since the financial crisis in 2008, international banking has attracted heightened interest from government authorities, researchers, financial practitioners, and other stakeholders. Perhaps no other sectors of the economy can better illustrate the potential benefits, as well as the perils of deeper integration and globalization than banking. It is also in this global financial crisis that China's banking sector stood out for its prudence and stability compared with the western counterparts. China, as the world's largest saver, has a major role to play in the global financial re-balancing toward emerging markets. Today, the emerging countries represent 38% of worldwide GDP but account for just 7% of global foreign investment in equities and only 13% of global foreign lending. The breaking down of regulatory shackles, and the breakthrough of information and technology in banking, combined with the integration of international financial markets and institutions, have created expanded opportunities and challenges for banking industry in both the developed economies and emerging ones to expand their markets on the global basis. The global banking arena provides abundant space for the proliferation of international banks, but at the same time can bring about more fierce competition.

To be more effective, capable, and active to hold a firm position in the global marching, many banks have adopted regulation changes, advanced banking technologies to approach further to international customers. On top of that, the most prevalent trend is the shift to digital transformation. A website is how the corporate represents itself in the digital world. It is just like a brand outlet but in the digital world. Still, some banks, especially the ones from developing economies, receive lesser than expected acceptance in the international level. Globalization of the banking industry does not only link to its financial performance and efficient operation. How it is perceived as a holistic corporate by the international community plays an equally important role. While the technological and regulatory innovation crushes the geographical barriers and national borders, the constraints due to different languages, cultures, and communication strategies still bring them limitations. The banking industry itself is created based on credibility, so if the bank cannot leave the impression as a trustworthy international, open, ethical institution to the global clients, how can it really go global? Given this, the online intercultural communication for the international banking industries plays a crucial role for sustainable overseas expansion. The challenge is that the clients today are no longer so myopia to chase the present small profits; instead, they are more finicky about the corporate image before they make the decision to purchase or invest, especially if that is a new bank brand from a different nation. In view of this, the globalization of banking industry is much more beyond the globalization of banking products, services, regulations, or standards, but also for the globalization of the corporate identity through the official websites.

## Literature review

### Corporate identity and corporate identity construction

Corporate identity (CI) is a concept with manifold interpretations. Traced back to the 1950s, this term was coined by graphic design consultants Lippincott and Margulies (1957). Evolved from the marketing domain, CI was mainly used for business people's commercial advertisement campaign and logo designing. Business practitioners and researchers found that there should be certain identities to express the corporate as an entity and make it clear to stakeholders. This process of conceptualizing the strategies, mission, and visions of the corporation employed *via* its business operations and productions is how the CI is explored (Melewar and Wooldridge, 2001).

There is still no universally agreed definition of CI due to the various constituent elements it entails. Some define it as “what an organization is” (Balmer and Greyser, 2002; Kitchen et al., 2013), “all corporate expressions” (Cornelissen and Harris, 2001), what a “company's ‘essence' is” (Olins and Council, 1978), or “what makes an organization unique and can incorporate the organization's communication, design, culture, behavior, structure, industry identity, and strategy” (Melewar, 2003; Melewar and Karaosmanoglu, 2006). There are also some scholars who try to drive away the “fogs” around the concept of CI to differentiate it from some similar concepts: CI is seen as something possessed by all organizations (Balmer and Greyser, 2003), to inform the corporate brand, albeit with some crossover (Balmer, 2001; Appel-Meulenbroek et al., 2010; Abratt and Kleyn, 2012; Kitchen et al., 2013), or a construct that is “multi-modal and multi-sensory” by bridging the concept of organizational identity (OI) and corporate identity (CI), thus using an all-encompassing definition of CI as the presentation of an organization to every stakeholder (Balmer, 2017).

Researchers or practitioners are more interested in the nature and feature: (1) CI is multi-dimensional and interdisciplinary: CI is to identify the essence of what the firm is and thus incorporates many unique characteristics of the firm such as history, philosophy, culture, communication, and the industry the firm operates in Melewar et al. (2005), and it is informed by various spatial/temporal dimensions from multidisciplinary perspectives in terms of comprehension and management (Balmer, 2001). (2) CI is dynamic: CI is no longer regarded as a “given” property to be represented and conveyed, but as “a multifaceted conceptualization of an organization's structure, roles, and values” (Elsbach and Bhattacharya, 2001). (3) CI is inseparable from the corporate personality of the organization (Melewar et al., 2005): it can never be separated from planned self-presentation of an organization, consisting of cues an organization gives *via* “its behavior, communication, and symbolism” (Riel, 1995). A new integrated strategic framework—“The corporate identity, total corporate communications, stakeholders' attributed identities, identifications, and behaviors continuum” (Balmer, 2017), was introduced. Recently, the research on how to manage the CI change process (Abratt and Mingione, 2017), the concept of corporate identity congruence (CIC) (Flint et al., 2018), and the relationship between CI and corporate sustainability (Simões and Sebastiani, 2017; Michaels and Grüning, 2018) also rise as the new trend.

A holistic classification of CI listed a few defining determinants, that is, corporate communication, corporate design, corporate culture, behavior, corporate structure, industry identity, and corporate strategy, each being categorized into a lower level to contain more determinants (Melewar, 2003). In theory, there exist three strands of thinking: (1) the interpretative view that a CI is an expression of the corporate personality; (2) the functionalist view that CI is an organizational reality; (3) the rhetorical view that CI is all the expressions of a company (Cornelissen and Harris, 2001). While in practice, CI mainly consists of three aspects: corporate design (e.g., logos and corporate colors), corporate communication (e.g., advertising and public relations), and corporate behavior (e.g., values and norms). Among all the determinants, Melewar (2003) regarded corporate communication as the first and foremost factor in CI determination. Communication refers to the aggregate of information an organization transmits to its stakeholders (Melewar and Karaosmanoglu, 2006). CI communication can enable organizations to obtain a positive image among its stakeholders, which in turn can generate a number of benefits including strong appeal to investors, better acceptance of products by consumers, and enhanced employee motivation.

### Role of websites in corporate identity communication

In the digital age, the global nature of the Internet has made it possible for people worldwide to visit any website wherever they reside and to participate online on an equal basis irrespective of national boundaries (Singh et al., 2005). Many corporations communicate with their public *via* online channels, such as the official website, the social media platforms, or news media online, among which a corporation's official website is often used for informing publics of its performance and other corporate-related information and for shaping a positive corporate image. Even in developing countries like China, companies, no matter large or small, have been found increasingly taking advantage of this new platform to deliver information, promote products, and engage stakeholders (Maynard and Tian, 2004). Corporate websites, with the easy access to customers and cost-effectiveness to corporations (Jayanti, 2018), serve as valuable sources of information and effective channels for communication (Buhalis and Law, 2008).

Although other communication channels can also be adopted, the official website still outstands for the following features: (1) corporate websites are increasingly becoming important channels for communications and are extensively relied upon by customers and stakeholders to search for information. (2) Corporate websites are chiefly maintained by companies for presenting the organization to external stakeholder groups, such as customers, investors, or the press. As such, the information disseminated on these platforms can be regarded as the intended information that companies wish to express to their stakeholders; (3) it is less likely that the information transmitted through this channel will be manipulated or filtered by gatekeepers because companies usually have direct control over their own websites (White and Raman, 1999). More importantly, companies utilize the official website for image building by addressing issues of corporate social responsibility (CSR), such as ecology, the environment, and education, and the CI constructed *via* websites asserts great impact on the CSR disclosure and implementation (Michaels and Grüning, 2018; Tourky et al., 2020). As the virtual storefront of the company, corporate websites have also been regarded as an important tool for public relations management (Park and Reber, 2008; Poppi, 2011). They should be integrated into the consistent branding strategy to create and communicate a coherent and strong image and to avoid any confusion and establish trust (Merrilees and Fry, 2002; Argyriou et al., 2006). Based on the quantitative research on the banking industry, a significant relationship between the CI and corporate websites has been identified. There is a need for firms' management to pay adequate attention to the nature of corporate identity that will be reflected by their websites in terms of culture, ethos, and philosophy guiding their operations and expectations (Balmer and Gray, 2000) and properly manage its communication strategy and information flow within it and among its numerous publics (Mutahi, 2021).

### Global corporate identity in intercultural communication

The global corporate identity construction in the global scale is much more complicated than in the domestic scope. A very effective practice is to use the international lingua franca “English” as a media in the international or English official websites for transmitting business information and corporate image. Many non-English speaking countries, like China or Korea, are also turning to this globalized trend. While the establishment of an English website can be viewed as a move toward globalization, it does not mean an unconditional adoption of global communicative models; nor does it mean a literal translation of Chinese websites (Gotti, 2011; Poppi, 2011). Some research analyzed how the companies with global reach develop their corporate identities through their official websites based on Aaker's brand personality dimensions (Abdullah and Seman, 2018). Today's notion of globalization, which emphasizes the coexistence and interpretation of the global and the local (Robertson, 1995; Wu, 2008), is not so binary opposite to the localization. Globalization does not necessarily lead to the suffocation of local cultures and cultural homogenization as some researchers predict (Hall, 1991), but, instead, may contribute to the reproduction of local cultures and facilitate the interaction between global and local cultures (Hongladarom, 1999; Wu, 2008). Intercultural communication, by its nature, is inclusive rather than exclusive. Likewise, glocalization emphasizes the dialectics and dynamics of “the global and the local, convergence and divergence, homogenization and heterogenization, universalism, and particularism” (Maynard and Tian, 2004). Therefore, the intercultural communication of the global corporate identity also entails the balancing between the local culture and global concerns.

A budding research interest influenced by this perspective about how the local and global elements are interacting in the corporate international websites is evolving. With regard to the various organizational culture worldwide, Geert Hofstede's cultural dimension, namely, the Power Distance, Uncertainty Avoidance, Individualism/Collectivism, Masculinity/Femininity, Long/Short-Term Orientation, and Indulgence/Restraint, gives us insights into understanding different cultures in intercultural communication for business organizations. This model has been widely applied in comparative cross-cultural research on global corporate branding and advertising (Albers-Miller and Gelb, 1996; Hsieh, 2002; Foscht et al., 2008; De Mooij and Hofstede, 2010; Pina et al., 2010), corporate identity, or image (Bravo et al., 2013; Klongthong et al., 2020). With the advent of the Internet and related networking and communication technologies, the impact of Hofstede's cultural dimension on the CRS communication on websites (Vollero et al., 2020), the global corporate websites (Robbins and Stylianou, 2002), or even the user-interface design (Marcus and Gould, 2000) becomes the main research focus.

## Research data and methodology

### Research aims and questions

With the rising importance of CI communication among businesses and the growing reliance on websites for information searches and communication (Bravo et al., 2012), an influx of studies is devoted to the analysis of corporate website in various industries such as institutions of higher education (Opoku et al., 2006; Ozturk, 2011), automotive business (Rolland and O'Keefe Bazzoni, 2009), and the aviation industry (Driver, 1999). Although there is some research on the banking sector, it is still confined to the term translation or content analysis approach. The research from the discursive construction and intercultural approach is far and few. This study focuses on the discursive construction of CI on the web by the top 10 commercial banks in China and the United States. China is the most representative oriental culture with the largest client base, while the United States is typical representation of the mainstream western culture. Those two research samples are of typical features of the collision or fusion of contrasting cultures in the same domain, which can conduce to “the specific identity a company is willing to convey to its multiple audiences” (Poppi, 2011). So, an intercultural discursive perspective can contribute to the understanding of what kind of identities they want to construct, as well as the cultural influences on the particular strategies they adopt. Therefore, the research questions guiding the aim of this research are as follows:

**RQ 1:** What are the recognizable corporate identities constructed by the commercial banks both by China and the US on the websites?
**RQ 2:** Are there any variations in the local cultural preference in the discursive corporate identity construction on the web between the banks of China and the US? If yes, how differently do they present themselves on the websites?
**RQ 3:** What kind of cultural dimensions can be identified in constructing the corporate identity?
**RQ4:** What strategies have been employed for the construction of the corporate identity?

### Data

The top 10 commercial banks in China and the United States are listed in Table 1 selected as the sample for this study according to the Global Market Intelligence Report of Wikipedia and the Ranking of the Federal Reserves released in 2021. The rationale for the selection and paring is based on the financial and social influence, client coverage, and representativeness. In the banking system, banks can be mainly divided into several types: the central bank, the commercial bank, the private bank, the investment bank, etc. Among those various types, the commercial banks are heavily regulated by the central bank, targeted to the public and small business with the vast variety of banking products and services, and might be open to public scrutiny once going public. As opposed to the investment bank, commercial bank is where most people do the banking, which makes itself exposed to the largest scope of stakeholders. Hence, the commercial banks are most concerned about the CI, since it will greatly influence the corporate credit, reputation, and performance.

**Table 1:** Top 10 commercial banks in China and the United States.

**Rank**	**China**	**US**
1	Industrial and Commercial Bank of China	JP Morgan Chase
2	China Construction Bank	Bank of America
3	Agricultural Bank of China	Wells Fargo
4	Bank of China	Citigroup
5	Bank of Communication	U.S. Bank
6	Postal Savings Bank of China	PNC
7	Industrial Bank	Capital One
8	China Merchants Bank	TD Bank
9	Shanghai Pudong Development Bank	Bank of New York Mellon
10	China Minsheng Banking	State Street

On the websites of those banks, the information is illustrated as a hypertext, which consists of massive information due to numerous hyperlinks and hierarchies. That is to say, the contents of one web page can include endless information with sub-section content linking to another web page. It is unrealistic to analyze all the information on the website, so this research will be confined to the “About us,” “Corporate Responsibility,” and “Investor Relations” sections. The “About us” section usually involves a company's self-introduction, including primarily the general introduction of the company and letters from the CEO (Poppi, 2011). The “Social responsibility” section conveys the information about how the company maintains a balance between pursuing economic performance and adhering to societal and environmental in operating. The “Investor Relations” serves as a bridge for providing market intelligence to internal corporate management. It aims to provide investors with an accurate account of company affairs for their buy and sell decisions. All those sections are crucial to construct a positive corporate image for the company and thus can be used as the data for this study. Only the text information will be selected to create two comparative corpora: the corpus of Chinese commercial banks (hereafter CB) and the corpus of the US commercial banks (hereafter UB). The CB corpus consists of 52693 tokens, while the UB corpus has 57022 tokens.

### Methodology

Different from the traditional way to construct the corporate identity such as logo designing, this research focuses on the corporate social responsibility (CSR)-based corporate identity. The CSR-based corporate identity is an approach to make for a socially responsible corporation through activities on the environment, clients, employees, communities, and all other members of the public sphere who may also be considered as stakeholders for a positive image. It was revealed that CSR communication did generate brand value and a good corporate reputation (Pérez and Rodríguez del Bosque, 2014). A company nowadays has to interact with various groups of primary and secondary stakeholders (e.g., investors, shareholders, employees, customers, and business partners) and social actors (e.g., local communities, media, NGOs, and lobby groups) (Paola Evangelisti and Giuliana Elena, 2011). This research will analyze the discursive construction of the corporate identity and dig out how the various stakeholders are addressed.

Given the exploratory nature of this objective, a corpus-assisted web-based discourse analysis approach is adopted. It relies on the corpus analytic tool Wmatrix 4.0, which is an online software for corpus analysis and comparison with annotation tools, standard corpus linguistic methodologies, and keywords method. It can undertake the automatic semantic analysis of text with the UCREL (Table 2) semantic analysis system (USAS) by classifying the English words into 21 major discourse fields based on Tom McArthur's Longman Lexicon of Contemporary English (McArthur, 1981) and revised in light of the practical tagging problems met in the course of the discourse.

**Table 2:** 21 major discourse fields in UCREL (http://ucrel.lancs.ac.uk/usas/usas_guide.pdf).

A	B	C	E
General and abstract terms	The body and the individual	Arts and crafts	Emotion
F	G	H	I
Food and farming	Government and public	Architecture, housing and the home	Money and commerce in industry
K	L	M	N
Entertainment, sports and games	Life and living things	Movement, location, travel and transport	Numbers and measurement
O	P	Q	S
Substances, materials, objects and equipment	Education	Language and communication	Social actions, states and processes
T	W	X	Y
Time	World and environment	Psychological actions, states and processes	Science and technology
Z			
Names and grammar			

For the sake of fine-grained analysis, the tagset is further sub-divided into 232 category labels. When the corpus data are uploaded, Wmatrix4.0 can automatically annotate and tag semantically to produce detailed information about the specific use of different semantic categories in this text. In addition, the software can also compare one corpus with another parallel corpus or a general reference corpus to identify key semantic categories (SMCs), that is, semantic categories which are statistically more frequent in the subject corpus than in the parallel or reference corpus. The semantic category analysis is very essential to identify the theme of the corpus. For the present research, both the CB and UB corpora will be compared to one general corpus—the British National Corpus (hereafter BNC) sampler written corpus. Then, the CB and UB corpus will be compared with each other so as to identify the cultural preference for constructing the CI.

## Results

After the semantic analysis of the CB and UB corpora, both the common and distinctive corporate identities are constructed through the corporate websites.

### Common global corporate identity construction

Table 3 illustrates the top 20 key SMCs of CB and UB after comparing with the BNC sampler written corpus, respectively. The semantic tags show semantic fields which group together word senses that are related by virtue of their being connected at some level of generality with the same mental concept. The LL means the log-likelihood value. The higher the LL value is, the more statistically significant it is. The semantic tags are ranked from high to low based on the LL value in Table 3.

**Table 3:** Top 20 key semantic categories of CB and UB (compared with BNC sampler written).

	**CB**			**UB**		
**Rank**	**Tagset**	**LL**	**SMCs**	**Tagset**	**LL**	**SMCs**
1	I1.1	5,015.86	Money and pay	I1.1	2,545.15	Money and pay
2	I1	2,529.72	Money generally	I2.2	1,554.47	Business: Selling
3	I2.1	1,501.08	Business: generally	I3.1	1,530.04	Work and employment: generally
4	I2.2	1,153.92	Business: Selling	I2.1	1,507.61	Business: generally
5	S7.1+	1,108.27	In power	S8+	1,230.99	helping
6	S5+	706.17	Belonging to a group	S5+	1,023.25	Belonging to a group
7	S8+	509.34	Helping	A6.3+	756.93	Comparing: varied
8	A2.1+	504.17	Change	I1	674.58	Money generally
9	I1.1+	391.15	Money: affluence	W5	487.03	Green issues
10	Z2	305.15	Geographical names	A1.8+	461.84	Inclusion
11	A15–	302.22	Danger	A9–	389.45	Giving
12	A5.1	288.89	Evaluation: good/bad	X9.1+	350.74	Able/intelligent
13	A1.8+	255.88	Inclusion	Y2	301.27	Information technology and computing
14	A9	227.48	Getting and giving; possession	X5.1+	270.80	Attentive
15	A5.1+++	191.20	Evaluation: good	X2.2+	233.02	Knowledgeable
16	W5	178.30	Green issues	A1.4	223.68	Chance, luck
17	A5.1+	191.20	Evaluation: good	G2.1	213.67	Law and order
18	I1.2	178.30	Money: debts	I1.3	201.01	Money: cost and price
19	Z3	175.96	Other proper names	X9.2+	197.98	success
20	I1.3+	170.05	Expensive	L1+	196.75	Alive

According to Table 3, some common SMCs are easy to identify in both the CB and UB corpora: I1.1 (Money and pay), I1 (Money generally), I2.1 (Business: generally), I2.2 (business: selling), S5+ (Belonging to a group), S8+ (Helping), A1.8+ (Inclusion), and W5 (Green issues). Those shared SMCs indicate that there are some similarities in the official websites of the commercial banks in both China and the United States. To have a better understanding of how those SMCs contribute to the construction of CI for those commercial banks, this research then examines some typical tokens of each shared semantic category.

Illustrated by Table 4, the key SMCs can be classified into three groups to profile some typical identities of the commercial banks. The first group, which includes I1.1, I1, I2,1, and I2,2, accounts for the first and foremost identity: the one that distinguishes itself with all other industries, namely, **the professional financial service provider**. Tokens of I1 and I1.1, such as finance, money, cashier, capital, funding, investment, ATM, cash, and portfolio, all illustrate the wide coverage of banking products or services that can be provided to customers or clients. Other tokens (I2.1 and I2.2), as the characteristics of the business entity, emphasize the relationship with the customers, clients, shareholders, or potential customers. This identity of professional financial institution also tries to close the tie with clients, shareholders, and other partners as illustrated by “markets,” “consumers,” “clients,” “client driven,” “shareholders,” “partnership,” etc., and to strengthen its image as a good service provider to all with the frequent use of words such as “serve,” “enable,” “serving,” and “facilitate.” As we can see from the following typical example:

(1) Surrounding its business transformation and the overall strategy of Internet finance, the bank actively practiced the concept of customer-oriented financial innovation, vigorously strengthened customer-centric, cross-profession and cross-channel integrated innovation and overall management.
(2) We can do this because of the strong company we have built: global in reach, local in execution, with an impressive set of products and capabilities and a steadfast commitment to provide exceptional client service.
(3) With serving the real economy as the foothold of operation and management, the bank has adhered to new ideas, new finance, and new services to support the supply-side structural reform and economic transformation and upgrading and to achieve its own healthy and sustainable development.

**Table 4:** Typical tokens of the shared SMCs in both CB and UB.

**Tagsets**	**SMCs**	**Example**
I1.1	Money and pay	Assets, investment, banking, shareholders, invest, profit, capital, Funding
I1	Money generally	Financial, commercial banking, bank, money, finance, financing, Cashier, ATM, cash
I2.1	Business: Generally	Financial services, firm, businesses, company, portfolio
I2.2	Business: Selling	Markets, consumers, clients, transaction, selling, client driven
S5+	Belonging to a group	Communities, corporate, team(s), together, network, member, partnership, organization, merge,
S8+	Helping	Serve, enable, service, provide, support, serving, helping, volunteer, encourage, promote, facilitate
A1.8+	Inclusion	Comprehensive, including, integrated, integration, inclusive, involving
W5	Green issues	Environment, environmental, eco, conservation, nature, conservancy

The second group, including Tagset S5+, S8+, and A1.8+, involves the relationship within the organization or within the communities, namely **the employee relation**. This can be indicated by tokens of Tagset S5+ such as “team,” “member,” “communities,” and “together,” which emphasize the team spirit and sense of belongs for the employees; tokens of Tagset S8+ such as “support,” “provide,” “encourage,” “helping,” and “promote” construct how supportive the corporates are to support the development of the employees and the community; tagset tokens of A1.8+ demonstrate the corporate identity as inclusive and open with words such as “integrated,” “inclusive,” and “incorporate.” As we can see from the following typical example:

(1) Diversity brings together people with unique perspectives, and inclusion creates opportunities for all individuals to contribute and work together to achieve success as a whole.
(2) Consistently provide honest and direct feedback; encourage people to say what is on their minds. Remove obstacles to facilitate the team's success.
(3) It aims at improving the professional competence of its staff and creating a positive, productive learning environment to raise awareness of the importance of learning among employees, to provide them with learning opportunities and to facilitate the learning of every employee.
(4) Excel by embracing an inclusive work environment and diverse teams.

Last but not the least, the third identity as an **environmentally and socially conscious corporate** is also created. Tagset W5 clearly makes the banks a corporate with environmental protection with words such as “environment,” “eco,” “conservation,” and “nature.” Environmental protection has been a universally acknowledged consensus for all human beings, as we can see from the presence of green issue tagset in both corporations. Besides, the image as a responsible, contributing, and giving corporate to the sustainable development for the diversified society is also constructed with the tagest of S8+ and S5+:

(1) Through these efforts, we are driving growth, investing in the success of our employees, helping to create job, developing communities, and fostering economic mobility and address society's biggest challenges while managing risk and providing a return to our clients and our business.
(2) We help strengthen the communities in which we live and work. Leverage our success to give back to the communities where we do business.
(3) Providing outstanding financial services—serving the customers, providing return to shareholders, realizing employee potential, and contributing to society “as the objective of the bank's social responsibility, the bank has always focused on the common needs of economic and social development and served the sustainable development of economic development and social progress.”

The common identities shared by the commercial banks in both China and the United States include the professional financial service provider, responsible corporation for employees and relevant communities, as well as a corporate with environmental and social consciousness. These identities correspond with the development and connotation of CSR. Carroll (1991) proposed the four-step pyramid of CSR, according to which a company's CSR activities comprise four segments: economic, legal, ethical, and philanthropic obligation. In an empirical study, it was discovered that economic responsibilities were given the most emphasis across the globe, followed by philanthropic activities in emerging nations such as Bangladesh, while legal responsibilities are in the third place and the ethical responsibilities come the last (Belal, 2008). Similarly, Strafella concluded the three waves of corporate social responsibility: wave (a) addresses shareholders, business partners, and customer relations; wave (b) concerns employee relations, such as health, safety, and training; wave (c) addresses community issues, environment, and philanthropy (Strafella, 2011).

Those corporate identities created as a common phenomenon are no coincidence. The economic-social-ethical multi-layer connotation of a company is a global trend influenced by the universal values of the international community. This framework applies to various nations or industries, but what distinguishes the commercial banking with other corporations is the money-related financial professionalism. Furthermore, as the commercial banks, the target market and clients determine the customer-oriented marketing and serving feature compared with other financial institutions. The inclusiveness is another manifestation of the abundant customer base and employee composition. It is also the strong connection to the public that make the commercial banks showing their responsible, reliable, and giving image for sustainable customer relationship.

### Distinctive corporate identity construction

After the analysis of the common key SMCs of CB and UB, this study further compares the two corpora to identify the distinctive key SMCs of each corpus, as demonstrated in Table 5.

**Table 5:** Top 20 SMCs in CB and UB compared with each other.

	**CB**			**UB**		
**Rank**	**Tagset**	**LL**	**SMCs**	**Tagset**	**LL**	**SMCs**
1	Z2	675.84	Geographical names	Z8	1,571.56	Pronouns
2	I1	227.32	Money generally	I3.1	424.23	Work and employment: generally
3	S7.1+	207.15	In power	A6.3+	179.55	Comparing: varied
4	I1.1	201.65	Money and pay	S2.1	124.51	People: female
5	N1	155.97	Numbers	A1.4	107.81	Chance, luck
6	Z5	108.77	Grammatical bin	S3.1	80.88	Personal relationship: general
7	A2.1+	73.09	Change	C1	76.10	Arts and crafts
8	N4	71.55	Linear order	A9+	68.61	Getting and possession
9	Z99	58.99	unmatched	W1	68.41	The universe
10	T1.3	49.15	Time: period	X2.3+	65.09	Learning
11	O4.1	48.42	General appearance and physical properties	A7+	61.60	Likely
12	Z3	47.28	Other proper names	A3+	59.65	Existing
13	N5+	45.36	Quantities: many/much	S2	54.01	People
13	A11.1–	40.95	unimportant	G3	49.59	Warfare, defense, and the army; weapon
15	S1.1.3+	39.96	Participating	A13.3	43.74	Degree: boosters
16	N5+	39.20	Evaluation: Good/much	S8+	41.72	Helping
17	I1.2	38.55	Money: debts	B2	40.14	Health and disease
18	M7	30.51	Places	G2.1	38.73	Law and order
19	O4.2	30.27	The media: newspapers etc.	K5.1	37.44	Sports
20	G1.1	29.27	Government	S4	35.54	Kin

The themes illustrated by the key semantic categories in each corpus are quite distinctive from each other, which can be a good proof for the preference in the CI construction in the two different cultures. This paper will analyze the distinctive cultural preference from three aspects:

#### Strategy

As for the strategy for corporate communication or publicity, the degree of proactivity varies from culture to culture. According to Strafella, “proactivity” means “the willingness of the company to meet expectations by anticipating issues and, most importantly, by shaping the agenda in advance” (Strafella, 2011). Corporates adopting **the proactive strategy** prefer to cause the positive image to be created rather than wait for the response from the public. Normally, those companies will take the initiative to transmit the information for the super positive image and avoid the negative parts. On the contrary, **the reactive strategy** is less active to impose positive tags to the company. The focus of those companies is to address the concerns of relevant stakeholders or to resolve some crisis or problems for their corporate image. By analyzing the typical tokens of the key SMCs, the strategy adopted by the UB and CB can be observed.

According to Table 6, which demonstrates the top 12 typical tokens of CB, the commercial banks in China prefer to emphasize the achievements they have made (A5.1, O4.2, and S1.1.3+). It is obvious that China's commercial banks do value the ranking or recognition from the outside sources such as the financial rating agency or the media (A5.1 and O4.2). This can be a good endorsement for their credibility. In particular, O4.2 is a unique semantic category in CB, since in the Chinese context, media is a major stakeholder in defining and promoting CSR (Lu, 2012). Besides, Chinese banks intent to construct a very reliable image by emphasizing their authoritativeness (G1.1). The connection to the government is a very typical feature for Chinese enterprises. The good relationship between the government and the commerce is a very useful backing for the development of a company in China. Apart from that, commercial banks in China intend to highlight their effort to adapt to the international level (M7) by transformations or reforms (A2.1+ and O4.1) or the participation to forums or conferences (S1.1.3+), because they do realize that the past economic pattern in China and the influence on the banking industry is a weakness for its global marching. It takes the proactive strategy to go beyond those potential negative respects by stressing the endeavors for internationalization and transformation. The frequent use of some highly positive adjectives (N5+) is another evidence of the proactive strategy to enhance the corporate image.

**Table 6:** Typical tokens of key semantic categories in CB.

	**CB**		
**Rank**	**Tagset**	**Semantic**	**Examples**
1	I1	Money generally	Financial, bank, finance, banks, insurance, commercial bank
2	S7.1+	In power	Management, board of directors, supervisors, board, committee, directors, control, leading
3	I1.1	Money and pay	Banking, shareholders, investment, asset, credit, funds, capital, securities, payment, card, fund, profit, deposits
4	A2.1+	Change	development, reform, transformation, become
5	N4	Linear order	First, previous, consecutive, the first,
6	O4.1	General appearance and	Structural, balance, conditions, image, structural, structured, characteristics
		physical properties	
7	N5+	Quantities: many/much	General, increase, increased, increasing, extensive, many, multiple, a number of, adequacy
8	S1.1.3+	Participating	Meeting(s), participate(d), collaboration, conference, parties, forum
9	A5.1	Evaluation: Good/bad	Rating, ranked, quality, standard, evaluation, appraisal
10	O4.2	The Media: newspapers etc.	Articles, magazine, correspondent, weekly,
11	G1.1	Government	Governance, state, duties, government, president, council, state-owned, authority, treasury
12	M7	Places	International, foreign, areas, settlement, cross-border, national, local

Whereas the key SMCs in UB illustrate that the strategy for commercial banks in the United States is on the other end in terms of the degree of proactivity. As revealed from Table 7, what the UB concerns most are those specific issues rather than the broad or ambitious visions presented in CB. A very big portion of the concerns for UB is about the employees or people, which demonstrates the employee-oriented or people-oriented corporate culture. Among the top 12 key SMCs, five are focused on the people: I3.1, S2.1, S3.1, S2, and S4. This is quite different from the CB corpus, which is more power or authority-oriented. Even as a financial institution whose major focus is about money or wealth, those American commercial banks still shed considerable light on the diversified groups of people, showing their respect for the females, employees, families of employees, or veterans. While commercial banks in China center on the general description of the achievements or efforts made by the banks in the financial filed, those in the United States are more inclined to address specific issues involving social progressing such as social diversity (A6.3+), arts and crafts (C1), warfare (G3), health and disease (B2), and financial education (X2.3+). Some negative aspects such as anti-corruption (G2.1) are also addressed.

**Table 7:** Typical tokens of key semantic categories in UB.

	**UB**		
**Rank**	**Tagset**	**SMCs**	**Examples**
1	I3.1	Work and employment: generally	Employee(s), works, career, workforce, working, role, volunteer, workplace
2	A6.3+	Comparing: varied	Diversity, diverse, variety, various, combination
3	S2.1	People: female	Woman, female(s), women-owned, girls-club, women-led
4	S3.1	Personal relationship: general	Meet, relationship(s), partners, associates
5	C1	Arts and crafts	Culture(s), designed, art(s), design, cultural
6	X2.3+	Learning	Learn, learning, learned
7	S2	People	People, gender, individuals, children, human, identity
8	G3	Warfare, defense, and the army; weapon	Military, veteran(s), officer, war
9	S8+	Helping	Help, services, serve, support, helping, benefits
10	B2	Health and disease	Health
11	G2.1	Law and order	Code, legal, laws, regulations, anti-corruption, attorneys, discipline(ed), rules, legislative
12	S4	Kin	Families, family, foster, engaged, parents, households, spouses, parental, maternity

#### Style

In corporate communication, a very crucial element to influence the effect of the corporate image is how the corporate positions itself in the distance with the putative audience. This can be regarded as the communication style in discourse, which varies from the personal to impersonal based on the dialogic distance. The public relation witnessed a transformation from the monologic persuasion to the dialogic communication with the public. Dialog is defined as “any negotiated exchange of ideas and opinions in public relations practice” (Kent and Taylor, 1998), and dialogic communication is an interactive and symmetrical way to leave a more ethnical impression to the public by the equal footing in the organization-public communication channel. This kind of communication style can be clearly identified according to the CB and UB corpora, as we can see that the most frequently used semantic category in UB is the pronoun than that in CB. If we dig further, among the pronoun, the frequency of “our” (1,432, 2.6%) and “we” (1,099, 2.0%) in UB is much higher than that in CB: our (245,0.52%) and we (193, 0.41%). The first-person pronoun, in particular, is “a strategy commonly used in corporate external communication to personalize the communication” (Degano, 2010). So is the second-person pronoun: you (67, 0.12%) and your (63, 0.11%) in UB and you (6, 0.01%) and your (5, 0.01%) in CB. While in CB, the proper names or the general business, financial institution names are a more overwhelming presence than that in UB: the token of bank (684, 1.45%) in CB and (89, 0.16%) in UB.

These statistics are very convincing evidence that commercial banks in the United States are more willing to shorten the distance between the corporations and the public, thus creating a personal style in CI construction by addressing the corporate and the public as the first-person pronoun and second-person pronoun, while commercial banks in China are prone to use the direct reference of the company to keep distance for respect. In other words, the commercial banks in the United States prefer to use the conversation style and personal communication while those in China are partial to the hierarchy style and impersonal communication.

#### Persuasion mode

Since the corporate identity construction is a way to persuade the public to recognize certain images or values of the specific company, this kind of communication can be regarded as a persuasion process. Then, the persuasion mode adopted by UB and CB can also be analyzed as the distinctive feature. As the devices in rhetoric, the persuasion mode, also referred as ethical strategies or rhetorical appeals, can be classified into etho, patho, and logos based on the speaker's appeal to the audience. Etho is the appeal to the authority or credibility of the presenter; patho is the appeal to the audience's emotion; logos is the logical appeal by describing the facts or figures to support the speaker's claim. Shifting the focus to the persuasive appeals of the discourse for corporate communication, the three layers of persuasion mode evolve into “credibility appeal, affective appeal, and rational appeal” (Hyland, 1998), respectively. Credibility appeal aims at convincing the audience that the presenter is “competent, trustworthy, authoritative, and honest persona” (Hyland, 1998). This can be realized by demonstrating the achievements in the professional field, introducing the bona fides from other established authorities, or showing the mastery of terminologies of the field, whereas affective appeal is realized by empathetical way and is most effective when the author demonstrates the agreement with the underlying value of the audience. So, if the concerns of the putative readers are addressed appropriately, or the positive future scenario is painted after following the course or action, then the affective appeal is quite powerful, while the rational appeal is normally used to describe facts or figures to support the writer's claim.

According to Table 6, the key SMCs in CB facilitate to transmit the trustworthy and credible corporate image by emphasizing the achievements and recognition by authority. The frequent use of the positive adjectives and the positive evaluation words, as well as the financial terminologies, all characterize the “credibility appeal” for CB. On the contrary, as the key SMCs in Table 7 demonstrate, UB takes into account the practical concerns of the various stakeholders and tries to resolve the problems with shared perspective of the public, which conforms to the typical “affective appeal.” As for the “rational appeal,” both UB and CB adopt this persuasion mode with concrete date, figure, graphics, or information to support the construction of the professional financial institution image.

## Discussion: Intercultural communication dimensional perspective

The above distinctive CI constructed in UB and CB can be explained from the intercultural communication approach. The national values differentiate from society to society and exert influential effect on the members and relate to their behavior. Hofstede's cultural dimension theory, created in 1980 by Dutch management researcher Geert Hofstede, provides a framework that can be used to understand the differences in culture across countries, the effects of culture on the values of its members, and to discern the ways that behavior is done across different cultures. Hofstede's theory aims to determine the dimensions in which cultures vary and is widely applied in fields like cross-cultural psychology, international management, and intercultural communication. Hofstede initially identified four value dimensions (Individualist/Collectivist, Power Distance, Uncertainty Avoidance, and Masculinity/Femininity). Additional research that used a Chinese-developed tool identified a fifth dimension: long-Term/Short-Term orientation (Bond, 1991) and a replication, conducted across 93 separate countries, confirmed the existence of the five dimensions, and identified a sixth known as Indulgence/Restraint (Minkov and Hofstede, 2012; Hofstede's Cultural Dimensions, 2021).

Based on the data and analysis above, it is obvious to distinguish the culture features of China and the United States. The Chinese Commercial Banks adopted the proactive strategy for corporate identity construction by emphasizing the achievements they have made, showcasing the ranking or recognition from the outside sources such as the financial rating agency or the media, highlighting the good connection with the government as well as the transformation and reform on financial mechanism. As for the communication style, the Chinese Commercial Banks are prone to take hierarchical and impersonal communication by using the direct references such as proper names and financial institution names more frequently than their counterparts of the United States so as to keep distance for respect. With regard to the persuasion mode, the Chinese commercial banks are more focused on the “credibility appeal” and “rational appeal” with the frequent use of the positive adjectives and the positive evaluation words, as well as the financial terminologies. Those are all typical representation of the high-context culture, namely, the high power distance where hierarchy is clearly established and executed in society without doubts or reason. Due to the Confucianism, the hierarchical concept has long been the backbone of the Chinese culture, which ensured the everlasting political stability and society governance in ancient times. The long interpersonal distance implies the authoritativeness and reliability due to the official endorsement beneath. The collectivism of Chinese culture unconsciously inclines to take more perspective from the institution level rather than the individual one. It does not mean that Chinese commercial banks care less about the individual, rather it is just taken for granted that each individual is included in the whole organization or institution. Besides, collectivism prefers conformity rather than novelty, which means high degree of uncertainty avoidance. This is fully manifested with the SMCs which indorse Chinese banks opt for stiff code of behaviors, guidelines, laws, and generally rely on absolute truth. In the dimension of masculinity and femininity, the more masculine society like China prefers achievement, heroism, assertiveness, and material rewards for success. So the achievements made by the banks or the positive recognition or awards from official departments prove to be the best manifestation of the credibility or authoritativeness of the institution. That is why in the high-context culture, the proactive communication strategy, the hierarchical and impersonal communication style, and credibility appeal persuasion mode are more likely to win the trust of the public, whereas the corporate identity construction strategy adopted by the commercial banks of the United States tells another story. The key SMCs of the US corpus are more people-oriented or employee-oriented, more concerned on the diversified and minority group, and more reactive in terms of the communication strategy. In regard to the communication style, the commercial banks of the United States position themselves in short distance with the putative audience with frequent use of the first-person and second-person pronounces. Regarding the persuasion mode, the commercial banks of the United States paid more attention to the practical concerns of the various stakeholders and try to resolve the problems with shared perspective of the public, which conforms to the typical “affective appeal.” These all prove that the commercial banks of the United States fit into the low-context culture, in which the fundamental philosophy is that power should be equally distributed. As a country founded based on the Declaration of Independence, the United States is a representation of the western country in pursuit of equality. The motivation for the British puritans shipping across the Atlantic Ocean is the willingness to break the hierarchy in both the political and religious field back in the UK. So in an individualist society like the United States, each individual's value matters. Even as an institution with lots of financial professionals, it still keeps short distance with the public with the dialogic communication style. It has loose ties that often only relate to an individual or the immediate family, that is why so many semantic categories about personal development or the family caring are found in UB, which is seldom in its counterpart, the collectivist Chinese culture, while a society with lower degree of uncertainty avoidance shows more tolerance for the differing thoughts or ideas, as we can see the semantic categories of diversity and inclusiveness in UB. For the dimension of masculinity or femininity, the more feminine society tends to show “a preference for cooperation, modesty, caring for the weak, and quality of life.” This distinction is well represented by the UB which care more for the disadvantaged and tries to promote the quality of life for both the employee and the public.

It is not fair to judge which communication strategy is more advanced or what kind of CI constructed through website is better. Intercultural communication itself entails distinctions and diversification. The different corporate identities constructed by the two groups of banks are just the reflection of the oriental and occidental culture. Locally, each communication strategy fits the cultural context. On the one hand, those differences should be understood and learned rather than refused or misunderstood. On the other hand, when certain business institutions want to be implanted into other soils, they should try to adapt and reconstruct in the new environment, even in the web space. So when the Chinese commercial banks going global want to win more trust among overseas market, the glocalization communication mindset is more than necessary.

## Conclusion and limitations

The research finds that both the common corporate identity and the distinctive ones are constructed through the corporate website discourse. The recognizable corporate identities constructed by the commercial banks both in China and the United States on the websites include the professional financial service provider, responsible corporation for employees, and relevant communities with environmental and social consciousness. In particular, the money-related financial professionalism showing their responsible, reliable, and giving image for sustainable customer relationship is most distinctive, while variations in the local cultural preference in the discursive CI construction on the web between the banks of China and the United States are manifested in the communication strategy, style, and persuasion mode: (1) The Chinese Commercial Banks adopted the proactive strategy for corporate identity construction, are prone to take hierarchical and impersonal communication style, and more focused on the “credibility appeal” and “rational appeal” in persuasion mode; (2) the commercial banks of the United States are more reactive in the communication strategy, position themselves in short distance with the putative audience in communication style, and conform to the typical “affective appeal” regarding the persuasion mode.

The common identities constitute the shared value or image for socially responsible corporate in the globe, while the distinctive ones are the representation of the peculiar high-context culture and low-context culture. The CI constructed by Chinese banks is just the reflection of the high-context cultural dimensions such as the high power, long interpersonal distance, lower degree of uncertainty avoidance, hierarchical and impersonal communication, higher degree of uncertainty avoidance, collectivism, and masculinity, whereas the CI constructed by the American banks is characterized with cultural dimensions like short distance, lower degree of uncertainty avoidance, as well as the individualism and femininity. The intercultural perspective shed a new light in the CI construction in the global scope. The globalization does not mean standardization or homogenization; instead, it is more inclusive and universal. The integration of the global communication methods and the local culture itself is a dynamic process of cultural adaptation and reconstruction. But when going global, the glocalization communication strategy is more than necessary if the corporations want to win more trust among overseas market.

To conclude, it is recommended that Chinese banking industry should try to shorten the cultural gap by adopting communication strategy in conformity with the local cultural psychology when going global rather than sticking to the domestic communication strategy. Due to the limited scale of the corpus, this research may not be so representative. So an extended research corpus with empirical research methodology such as questionnaire or interview will be the potential for future research.

## Data availability statement

The original contributions presented in the study are included in the article/supplementary material, further inquiries can be directed to the corresponding author.

## Author contributions

HF: conceptualization, methodology, software, investigation, formal analysis, and writing-original draft. HZ: research design, administration, funding acquisition, writing-reviewing and editing, and final approval of the version to be published. Both authors contributed to the article and approved the submitted version.

## Funding

This work was supported by Zhejiang Provincial Philosophy and Social Science Planning Project (grant number 20NDQN291YB), Zhejiang Gongshang University-Foreign Language and Literature first-class discipline of Zhejiang Province (Class A) (grant number 2020YLZS10), 2021 Domestic Visiting Scholars-Teacher Professional Development Program (grant number 1070KU2222003), and 2021 Undergraduate Teaching Reform Project of Zhejiang Gongshang University (grant number 1070XJ2921060).

## Conflict of interest

The authors declare that the research was conducted in the absence of any commercial or financial relationships that could be construed as a potential conflict of interest.

## Publisher's note

All claims expressed in this article are solely those of the authors and do not necessarily represent those of their affiliated organizations, or those of the publisher, the editors and the reviewers. Any product that may be evaluated in this article, or claim that may be made by its manufacturer, is not guaranteed or endorsed by the publisher.
